# High-Resolution Manometry—Observations After 15 Years of Personal Use—Has Advancement Reached a Plateau?

**DOI:** 10.1007/s11894-020-00787-x

**Published:** 2020-08-07

**Authors:** Rami Sweis, Mark Fox

**Affiliations:** 1 Gastrointestinal Services, University College London Hospital, London, UK; 2 Division of Gastroenterology and Hepatology, University Hospital Zürich, Zürich, Switzerland; 3 Digestive Function: Basel, Laboratory and Clinic for Motility Disorders and Functional GI Diseases Center for Integrative Gastroenterology, Klinik Arlesheim, Arlesheim, Switzerland

**Keywords:** High resolution manometry, Dysphagia, Impedance, Achalasia, Reflux disease, Dysmotility

## Abstract

**Purpose of Review:**

In the absence of mucosal or structural disease, the aim of investigating the oesophagus is to provide clinically relevant measurements of function that can explain the cause of symptoms, identify pathology and guide effective management. One of the most notable recent advances in the field of oesophageal function has been high-resolution manometry (HRM). This review explores how innovation in HRM has progressed and has far from reached a plateau.

**Recent Findings:**

HRM technology, methodology and utility continue to evolve; simple additions to the swallow protocol (e.g. eating and drinking), shifting position, targeting symptoms and adding impedance sensors to the HRM catheter have led to improved diagnoses, therapeutic decision-making and outcomes.

**Summary:**

Progress in HRM persists and shows little sign of abating. The next iteration of the Chicago Classification of motor disorders will highlight these advances and will also identify opportunities for further research and innovation.

## Introduction

The oesophagus is a muscular tube that transports food and fluid from the mouth to the stomach. It is bordered proximally by the upper oesophageal sphincter and distally by the lower oesophageal sphincter (LOS). The oesophago-gastric junction (OGJ) is a physiological barrier comprised of an overlap between the LOS and the crural diaphragm, which permits passage of swallowed material and selectively allows venting (belching) of swallowed air while reducing reflux of other gastric contents. Abnormal pressure activity alone is rarely symptomatic. Instead, oesophageal symptoms such as dysphagia, regurgitation, heartburn or chest pain (and also mucosal damage) occur when abnormal oesophageal motility disrupts bolus transport and reflux protection [1–4].

Patients with oesophageal symptoms who seek medical advice are often referred for an endoscopy to exclude mucosal or structural pathology. In the absence of infectious, inflammatory or neoplastic disease, and in the event of failure to respond to empirical therapy (e.g. acid suppressant medicines), guidelines recommend investigations of oesophageal physiology in the form of manometry and ambulatory reflux testing [5••, 6••, 7]. Advances in the technology, methodology, interpretation and reporting associated with these investigations have progressed at a rapid rate over the last 15 years.

### Manometry

Manometry is the mainstay of investigating disorders of oesophageal function. Technological progression of this method, including increasing numbers of pressure sensors and integration of impedance sensors into catheters, has yielded important new insights into pathological mechanisms of disease. These insights in turn have informed diagnostic classification and opened up therapeutic options that, until recently, simply were not possible.

Prior to the introduction of high-resolution manometry (HRM) into clinical practice approximately 15 years ago, manometry was limited to 4–8 sensors and presented the occlusive pressure, duration and velocity of peristaltic contractions as “line plots”. The clinical application of this technique was limited by subjective interpretation of findings and relatively poor sensitivity for motility disorders [1]. HRM—with at least one sensor every 2 cm along the length of the oesophagus combined with spatiotemporal, topographical representation of the pressure data (“Clouse plots”)—provides a compact, visually intuitive representation of oesophageal pressure activity from the pharynx to the stomach, which is significantly easier to interpret, and learn, than the more abstract information provided by “line plots” [8, 9].

**Fig. 1:**
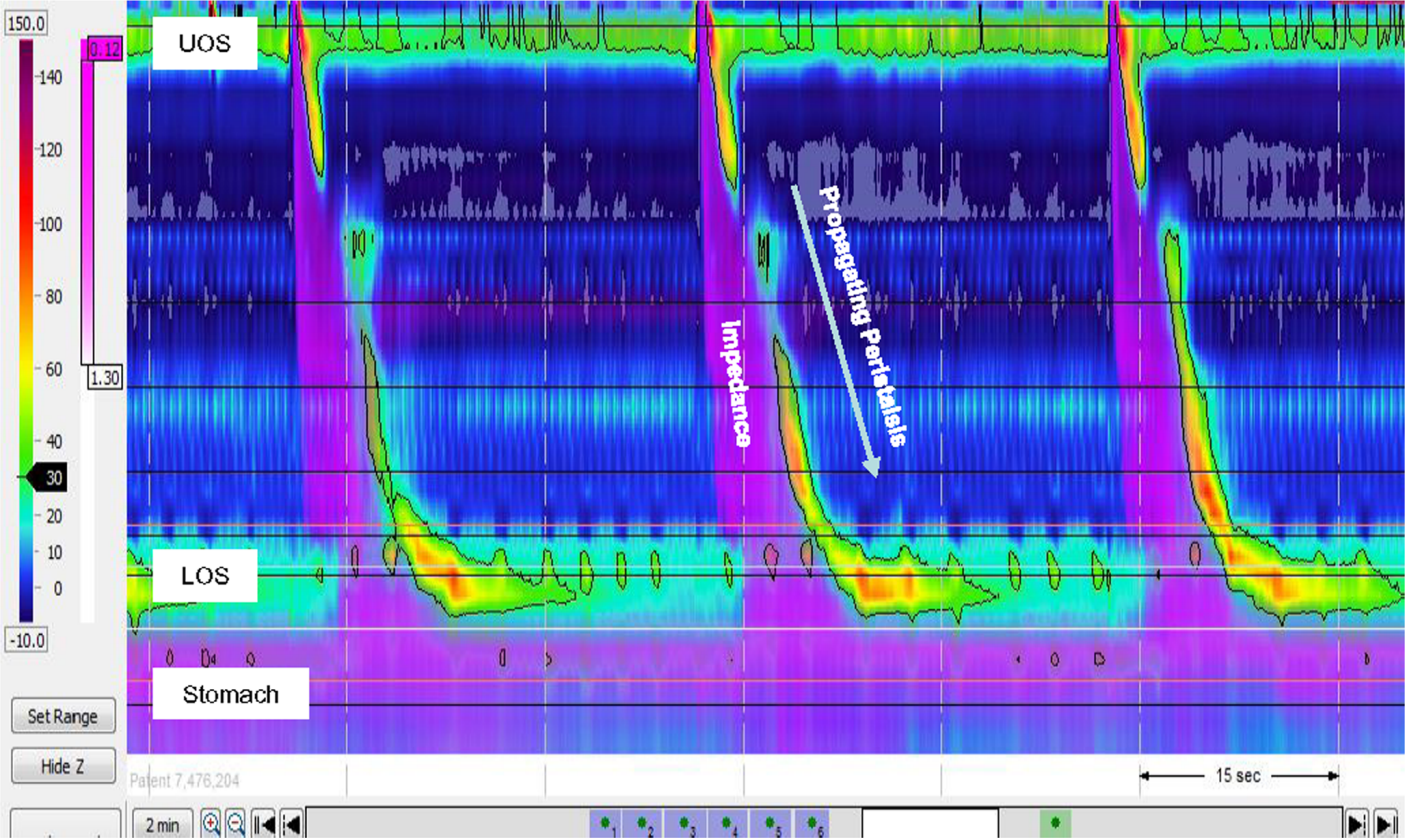
High-resolution manometry pressure data is presented as a spatiotemporal plot with overlay of the impedance trace. The spatiotemporal plot depicts oesophageal pressure activity from the pharynx to the stomach with pressure sensors spaced at < 2-cm intervals. Time is on the x-axis and distance from the nares is on the y-axis. Pressure is represented as changes in colour (legend left). The impedance trace (pink) provides a direct assessment of bolus transport down the oesophagus and into the stomach that confirms the functional effects of the pressure activity, similar to a barium swallow but without the radiation. (UOS: Upper oesophageal sphincter; LOS: Lower oesophageal sphincter)

Two main HRM technologies are available. Water-perfused systems are comprised of a collection of thin (micro-capillary) plastic tubes, each with a small hole that opens at various points within the oesophageal lumen, which interact with the mucosal wall such that changes in pressure can be monitored by an external transducer located at the perfusing pump. By contrast, solid-state systems involve miniature, often circumferential pressure sensors arranged in sequence along a single catheter. The latter system can be combined with multiple impedance sensors to provide a simultaneous and integrated assessment of pharyngeal and oesophageal motility and function (i.e. bolus transport, reflux) in real time (Fig. 1).

With the introduction of computerized software algorithms, metrics can be derived from the complex pressure (and impedance) data that determine the success or failure of bolus transport through the oesophagus and sphincters [10]. An uninterrupted, well-coordinated peristaltic contraction generates a “positive pressure gradient” that is likely to clear the bolus [1, 8] (Fig. 1). If this sequence is interrupted due to weak, exaggerated, simultaneous or obstructive pressure activity, it will likely lead to disturbed bolus transport; if interrupted sequences are repeated, food and fluid will be retained in the oesophagus, usually generating symptoms [8].

A number of proprietary software packages have been developed that, in principle, can process and interpret HRM data, providing a diagnosis “at the click of a button”. Automated analysis technology is most adept at defining normal studies and disorders like “classic achalasia” that are defined based on specific metrics (e.g. integrated relaxation pressure) consistent across a series of single water swallows. However, oesophageal motility can be highly variable, and expert opinion is still required to interpret findings and diagnose pathology, especially if it is present only intermittently or apparent only when motility is assessed in particular circumstances, for example, during rapid drinking or a solid test meal. Still, innovation in software algorithms is catching up with clinical practice. Integration of novel parameters and manometric benchmarks that define clinically relevant, symptomatic motility disorders continue to improve automated diagnostic accuracy with every iteration.

### Chicago Classification

Advances in HRM technology have led to a deeper understanding of normal oesophageal function as well as the discovery of novel disease processes, such as subtyping achalasia. When HRM first emerged however, analysis and interpretation were inconsistent among various institutions, and there was a lack of clarity with regard to what defined true pathology versus what was at risk of over-interpretation. It soon became clear that there was an unmet need for standard operating procedures for HRM performance and interpretation [1, 8]. To this end, the HRM Working Group was created, comprising clinicians and scientists who had developed and validated the technology. This process produced a new classification of oesophageal motility disorders that was coined the Chicago Classification, in recognition of the important work led by John Pandolfino and Peter Kahrilas at the Northwestern University. This system has been informed by the results of clinical studies and refined over time. The first three iterations of the Chicago Classification have been cited > 2000 times, and at the time of writing this review, version 4.0 is in preparation.

**Fig. 2:**
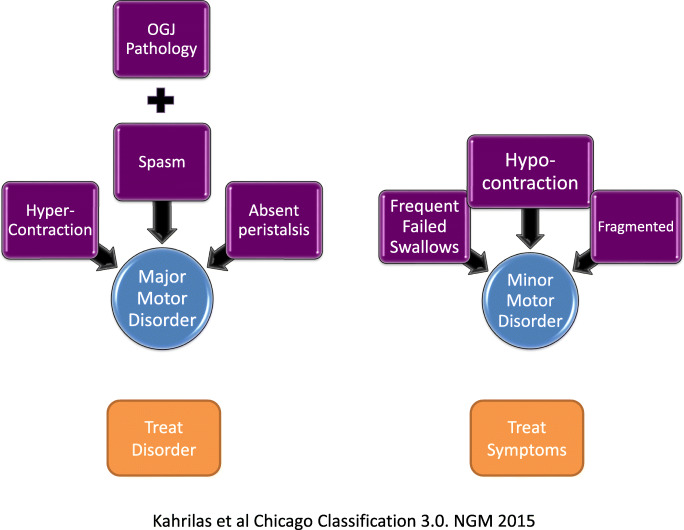
Chicago Classification of motility disorders is a hierarchical breakdown of abnormalities of oesophageal function based on high-resolution manometry analysis of 10 swallows of 5 ml of water. Major motility disorders are never found in healthy individuals, are commonly associated with impaired bolus transport and often lead to symptoms. Pathology of the OGJ is always considered first. Peristalsis abnormalities of Minor motility disorders can also be found in asymptomatic individuals and be a variant of normal

The cornerstone of this classification is its hierarchical nature [11]. Pathology within the OGJ is considered first based on the biophysical principle that obstruction at this level has a greater impact on bolus transport than abnormal motility within the body of the oesophagus (Fig. 2). In addition, there is a clear distinction between motility anomalies that can be seen in healthy subjects and might not be the source of symptoms (minor motor disorders) and those that are almost never seen in health are very likely to interrupt bolus transport and often lead to symptoms (major motor disorders). In the former category, if therapy is required, then empirical symptomatic management is applied. By contrast, in the latter category, there is a clear rational to direct treatment at the disorder itself [12, 13]. Indeed, studies have suggested that finding normal motility or a minor motor disorder is a good prognostic indicator [14]. Of the 98 patients with minor motor disorders, Ravi et al. showed that at 5 years, 70% were asymptomatic having exhibited spontaneous improvement [14].

The mechanism by which motility disorders are defined continues to progress. Novel parameters continue to be developed which will help to define new disease entities, subtype existing pathology, and, crucially, reaffirm what is normal. The next iteration of the Chicago Classification will encompass some of the new concepts, but with the integration of impedance into HRM catheters, it is clear that advances in the technology, analysis and our understanding of underlying pathophysiology will continue to grow.

Compared with “conventional manometry” based on data obtained from 8 sensors, HRM technology, combined with presentation of pressure data as Clouse plots, has been shown to improve inter-observer agreement for diagnosis of clinically relevant motility disorders [9, 15–17]. Additionally, case series and a large prospective trial have confirmed that this approach increases diagnostic yield (and accuracy) for achalasia and other major motility disorders [18, 19]. Despite these advances, HRM studies still have limitations. For example, at least 20% of patients with swallowing disorders have normal findings on HRM [8], and conversely, abnormal motility detected during single water swallows is only weakly associated with symptoms or outcomes [20, 21]. These limitations may be due in part to investigation in a non-physiological position, as the standard is to measure patients while supine in order to exclude gravity; people rarely drink or eat lying down, however, and investigations in the upright position are easier to perform and favoured by patients [22, 23]. Additionally, symptoms are much more likely to occur with solid rather than liquid swallows, and so the use of protocols that assess only single water swallows will miss clinically relevant, symptomatic pathology that may be apparent only with ingestion of a solid bolus [24, 25].

### Testing Protocol

“Conventional” manometry studies were traditionally performed using small volume (5 mL) water swallows in the supine or left lateral position [26]. Studies performed to validate HRM also employed small volume water swallows, and this protocol has endured as the foundation of the Chicago Classification. However, swallowing small volumes of water does not represent normal behaviour, very rarely reproduces symptoms and may lack sensitivity for clinically relevant disorders. To address this limitation, there has been a growing interest in performing studies that include a variety of standardized, “adjunctive” (also known as “provocative”) tests in attempt to highlight abnormal motility and reproduce symptoms. These include multiple rapid swallows, rapid drink challenge, ingestion of viscous material (apple sauce, yogurt), single solid swallows (bread, marshmallow, biscuit) or asking the patient to consume a standardized test meal.

The inherent logic in using such techniques has been widely accepted, such that some form of adjunctive swallowing is now commonly used in routine clinical practice in many oesophageal units. A recent international survey found that, out of 91 oesophageal centres around the world, 77% included drinking larger volumes of water, 63% included single solid/viscous swallows and 18% included a test meal in routine oesophageal physiology testing [27•]. The introduction of adjunctive testing is arguably the most important advance since the development of HRM. These tests will be included in the upcoming Chicago classification version 4.0 protocol and remain a growing area of interest and research.

## Patient Positioning

Until 10 years ago, manometry was most often performed with water perfused catheters. These investigations were performed in the supine or left lateral position to avoid hydrostatic artefacts related to the weight of the water in the catheter [8]. Solid-state HRM catheters acquire reliable pressure data in any position [8]. A recent prospective study in a large cohort of consecutive patients demonstrated diagnostic agreement in the supine and upright positions in approximately two-thirds of subjects, with discordant findings being the most frequent for ineffective oesophageal motility (a minor motor disorder); this discrepancy could easily be corrected by applying position-specific diagnostic thresholds [28••]. Concordance increased to nine in ten when only major motility disorders were considered; however, consistent with work from the Chicago group [29•], there was a high prevalence of false positive diagnoses of oesophago-gastric junction (OGJ) outflow obstruction in the supine compared with the upright position. It was concluded that HRM studies can be performed in either position, using appropriate reference values; however, if unexpected findings are observed, then swallows should also be evaluated in the alternative position (and/or with adjunctive tests) [28••].

## Multiple Rapid Swallows (MRS)

**Fig. 3:**
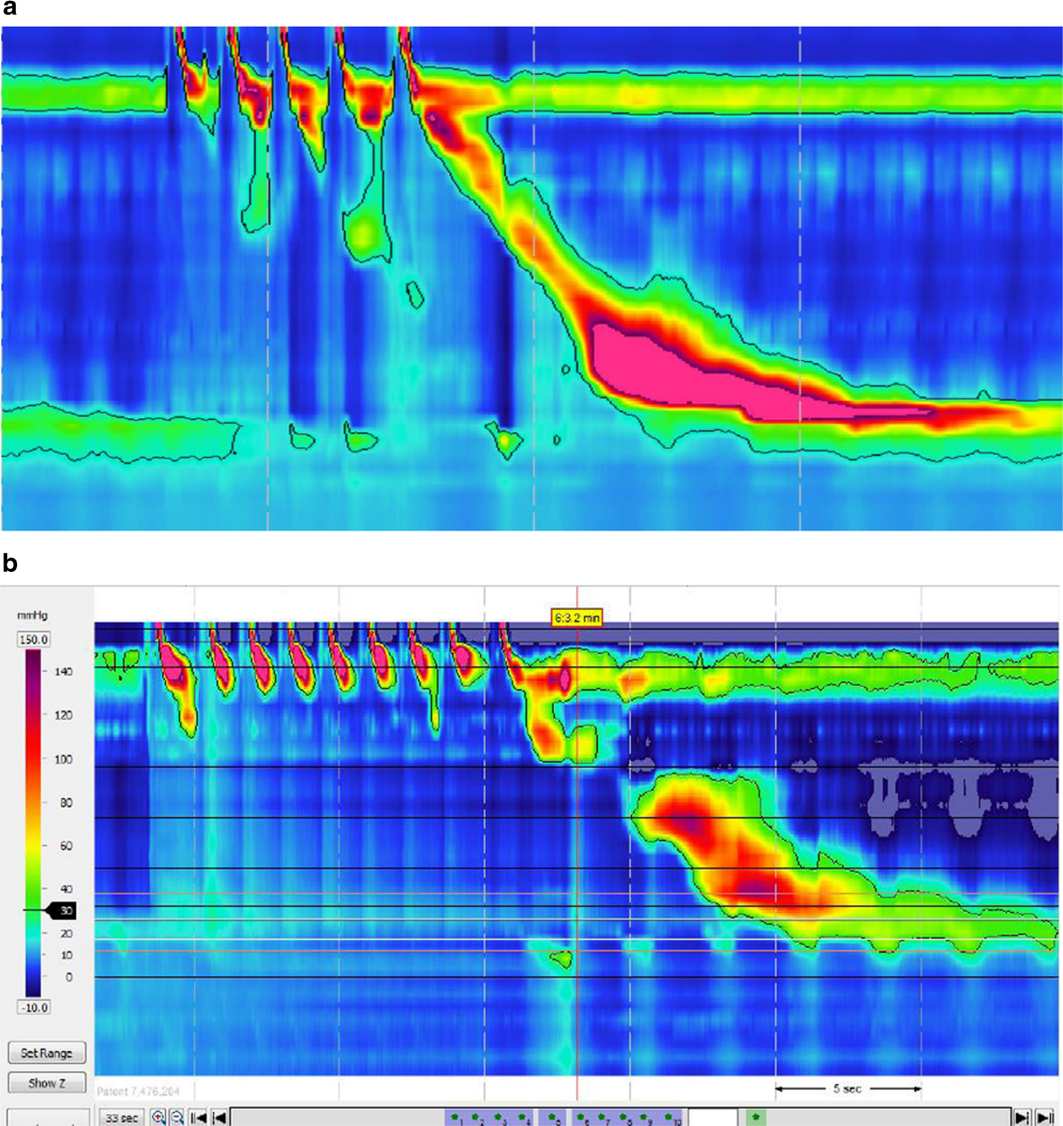
**a** and **b**. Multiple rapid swallow (MRS)—5 aliquots of 2 ml of water are given through a syringe such that the water is swallowed in succession. Rapid drink challenge (RDC)—200 ml of water is drunk in one go through a straw. In health, rapid swallowing leads to deglutitive inhibition whereby oesophageal peristalsis is inhibited with concomitant relaxation of the LOS. After the last swallow of the series, a pronounced clearing contraction commonly follows

Repetitive swallowing inhibits oesophageal body motility and enhances relaxation of the LOS, a phenomenon known as deglutitive inhibition. Thereafter, the presence or absence of a clearing “post-contraction” may provide information about neuromuscular function. For the multiple rapid swallows (MRS) test, the operator repetitively “introduces” 2-ml aliquots into the patient’s mouth to produce a series of five swallows in quick succession [24, 30–33, 34••]. MRS does not fill the oesophagus but rather enhances deglutitive inhibition during swallowing and, in many healthy subjects, can induce a powerful contraction at the end of the series (Fig. 3a). The presence of this “augmented post-contraction” is a marker of peristaltic contractile reserve in patients demonstrating ineffective oesophageal motility with single water swallows [35]. The absence of this contractile response following MRS in preoperative assessment has been associated with the occurrence of dysphagia following anti-reflux surgery [31, 36]. Although MRS is a relatively simple manoeuvre to perform, the resulting contractile response is variable, and the test should be repeated three times to provide reliable results. Additionally, normal values from a large cohort of healthy subjects have not yet been published.

## Rapid Drink Challenge (RDC)

Drinking larger volumes of fluid is a simple adjunctive test that can add important information to the motility assessment. The rapid drink challenge (RDC) involves drinking 100–200 ml of water by a series of small swallows, often through a straw. The RDC fills the oesophagus with water, which facilitates the detection of OGJ outflow obstruction (Fig. 3b) not reliably detected by single water swallows [34, 35]. The results are highly reproducible, and normal values have been established in large, prospective case series [34••]. Clinical studies have also confirmed that RDC can increase sensitivity for disorders of OGJ function and resolve diagnostic discrepancies (e.g. false positive diagnosis of OGJ outflow obstruction) [28]. In particular, inclusion of RDC in patients with dysphagia and suspected achalasia who exhibited absent motility but had a normal integrated relaxation pressure (IRP) on single water swallows confirmed the diagnosis in 79% of patients, all of whom responded on subsequent treatment to relieve obstruction to the same degree as those with standard achalasia [37•].

## Single Solid Swallows and Test Meals

Inclusion of single solid swallows and test meals have been known to provoke underlying oesophageal motility disturbances and symptoms in patients with suspected dysmotility since the 1980s [38]. Including bread as a test bolus during conventional manometry led to enhanced contractions in healthy subjects; however, the occasional presence of oesophageal pressurization and non-peristaltic contractions made interpretation difficult [39]. HRM greatly facilitates the assessment of oesophageal function, and several studies have been conducted in an attempt to understand, standardize and protocolize the technique [25, 40].

The healthy oesophagus responds to the challenge of high bolus consistency by enhancing the coordination and vigour of contractions to overcome increased viscous resistance to bolus passage through the oesophagus and EGJ [25, 41, 42]. As with MRS, the inclusion of solid swallows can reveal normal contractile reserve in patients with ineffective, weak or absent contractions with single water swallows. On the other hand, persistence of ineffective motility with solid swallows can confirm the diagnosis of a clinically relevant, symptomatic ineffective motility disorder. This is important in patients with swallowing disorders as well as gastro-oesophageal reflux disease who are considering anti-reflux surgery [24]. In published studies, the contractile response to solid swallows differentiated patients with non-erosive reflux disease from those with mucosal disease (reflux oesophagitis or Barrett’s metaplasia) significantly better than single water swallows [43, 44•]. Such findings confirm that mucosal disease develops in reflux disease as a consequence of inefficient oesophageal clearance.

HRM findings are more complex during ingestion of a meal than during single water swallows, and it is important to avoid over-interpretation in the clinical setting. In the last 4–5 years, standard operating procedures for the performance and analysis of this data have been introduced and validated. It has been shown that the results are reproducible and increase diagnostic yield for clinically relevant motility disorders. One early HRM study showed that when patients with reflux-like symptoms were compared with healthy subjects, the addition of a standardized meal led to a change in manometric diagnosis in 67% of patients, and to a change in the clinical diagnosis in 39%. In addition, the results appeared to guide effective management when outcomes were reviewed at 2-year follow-up [24].

Recent standardization of the meal protocol with presentation of normal values from 72 healthy subjects has facilitated this test being used in clinical practice [45••]. In a study of 750 patients, inclusion of a standardized test meal with the HRM protocol doubled the diagnostic yield of major motility disorders and identified the cause of oesophageal symptoms in more than two-thirds of patients [46••]. In another study of patients who presented with dysphagia following anti-reflux surgery, compared with single water swallows, the inclusion of a test meal detected more patients with symptomatic outlet obstruction (30% vs. 70%), many of whom subsequently responded to pneumatic dilation [47]. Additionally, continuing observations into the postprandial period can identify functional disorders such as rumination syndrome and supragastric belching [48, 49]. The enhanced diagnostic accuracy and clinical relevance of HRM findings have led some groups to propose using the solid meal as the standard test in place of single water swallows. The most appropriate protocol for HRM studies is the subject of ongoing research and is likely to entail a combination of techniques tailored according to the individual circumstance and presentation.

### Impedance Manometry

Impedance is defined as the opposition to current flow; in the presence of an electric current, impedance is inversely related to the conductivity of the surrounding medium and is dependent on the cross-sectional area of a cylindrical structure. If impedance sensors are placed on a catheter within the oesophagus, impedance becomes an inverse measurement of electrical conductance of contents within the lumen and is dependent upon the cross-sectional area of oesophageal lumen as well as the ionic composition of its contents. A bolus with a high ionic content (e.g. swallowed food/fluid bolus or refluxate) leads to high conductivity and a low impedance measurement, while a bolus with no ionic content (e.g. swallowed air or belch) has no conductivity and produces a high impedance measurement.

When combined with manometry, impedance can measure the content and direction of bolus movement within the oesophagus; impedance sensors use differences in resistance to alternating current between mucosa, liquid and air to determine bolus consistency [50] as well as the direction of bolus transit, without exposure to radiation. This is particularly relevant for those with dysphagia and normal manometry, who can comprise up to 50% of referrals for oesophageal physiology testing with swallowing problems [51]. On its own, HRM can only predict bolus transport by inference. As the relationship between motility and transit is complex, Bogte et al. showed that HRM-based metrics often poorly predict bolus transit failure, which is a common source of symptom generation [52].

Technological advances have integrated impedance with HRM, leading to a combined technique frequently dubbed HRIM (high-resolution impedance manometry). This technique was found to be sensitive and specific for the detection of pharyngeal motor disorders as a means of stratifying aspiration risk [53]. This novel technology has subsequently been adapted to assess bolus flow through the oesophageal body and across the OGJ [54]. Automated impedance-manometry analysis (AIM) combines information with regard to oesophageal pressure, bolus direction and flow; and intercalates key points within the impedance and manometric recordings to define subtle changes in oesophageal function not appreciable with standard analysis. In addition, viscous swallows can improve the diagnostic yield for detecting abnormalities of motor function [55]. Commercially, HRIM catheters are available with up to 18 impedance sensors. With all of these catheters, impedance colour contour-plot views are routinely provided such that bolus success or failure can be visible with every swallow to aid diagnosis (Fig. 1).

Studies continue to identify novel parameters which are proving to be useful in defining motility disorders in patients with non-obstructive dysphagia. Whether the addition of impedance to HRM and/or the inclusion of these novel parameters will aid diagnosis and guide management is a topic of ongoing research.

## Conclusion

Advances in technology, protocols, and clinical experience with HRM have not plateaued but rather have accumulated dramatically over the past 10–15 years. Studies have confirmed the superiority of this technology over conventional manometry, and new insights into the causes of oesophageal symptoms and disease have been won. Outcome studies have begun to show how this increase in knowledge translates into improved patient care, including introduction of novel therapies. Publication of the next iteration of the Chicago Classification will cement these advances, but there is no question that new questions will continue to emerge, generating additional opportunities for research and innovation.
